# The Obligate Human Pathogen, *Neisseria gonorrhoeae,* Is Polyploid

**DOI:** 10.1371/journal.pbio.0040185

**Published:** 2006-05-30

**Authors:** Deborah M Tobiason, H. Steven Seifert

**Affiliations:** 1Department of Microbiology-Immunology, Feinberg School of Medicine, Northwestern University, Chicago, Illinois, United States of America; University of California DavisUnited States of America

## Abstract

We show using several methodologies that the Gram-negative, diplococcal-bacterium Neisseria gonorrhoeae has more than one complete genome copy per cell. Gene dosage measurements demonstrated that only a single replication initiation event per chromosome occurs per round of cell division, and that there is a single origin of replication. The region containing the origin does not encode any genes previously associated with bacterial origins of replication. Quantitative PCR results showed that there are on average three genome copies per coccal cell unit. These findings allow a model for gonococcal DNA replication and cell division to be proposed, in which a minimum of two chromosomal copies exist per coccal unit within a monococcal or diplococcal cell, and these chromosomes replicate in unison to produce four chromosomal copies during cell division. Immune evasion via antigenic variation is an important mechanism that allows these organisms to continually infect a high risk population of people. We propose that polyploidy may be necessary for the high frequency gene conversion system that mediates pilin antigenic variation and the propagation of N. gonorrhoeae within its human hosts.

## Abstract 2

The authors provide evidence that the bacterium *Neisseria gonorrhoeae* contains on average three completely replicated genome copies per cell.

## Introduction

Bacterial chromosome replication results in duplication of genomic DNA for separation into daughter cells during cell division. Two of the best characterized bacterial replication systems are those of Escherichia coli and *Bacillus subtilis.* Both organisms have their genome located on a single chromosome. Under conditions of slowed growth, chromosomal DNA replication is completed prior to cell division, and the bacteria are transiently diploid, containing two fully replicated chromosomes. During rapid growth, the time between rounds of cell division can be less than the time required to replicate the chromosome; therefore, cell cycles can overlap, resulting in chromosomes that have completed one round of replication but have active replication forks at the time of segregation into daughter cells [ 1]. The presence of multiple pairs of replication forks on a single chromosome explains why exponentially growing E. coli cells can divide every 20 min, yet take 40 min to replicate their chromosome [ 2, 3]. Upon entry into stationary phase, cell division essentially stops, initiation of replication ceases, and all replication forks finish DNA replication resulting in multiple fully replicated chromosomes [ 4]. Once these cells return to a replicative growth phase, they are believed to return to a single chromosome per cell, which resumes active replication [see reviews 3,5]. Most bacteria behave as genetically haploid organisms and are assumed to essentially carry a single genome copy per cell.

Members of some bacterial genera have their genome distributed over several chromosomes, including *Vibrio, Brucella, Agrobacterium, Burkholderia, Pseudomonas, Rhizobium,* and *Rhodobacter* [ 6– 12]. Even though these bacteria have multiple chromosomes per cell, the chromosomes each contain different sets of genes. Therefore, these species have a single genome copy per cell and behave as haploids [ 13].

There are several bacteria with their genome located on a single or multiple genetic elements that contain more DNA per cell than a single genome copy could explain, and these bacteria have been proposed to be polyploid. These organisms include the radioresistant bacterium *Deinococcus radiodurans,* which has been reported to carry four genome copies per cell in stationary phase and up to ten copies per cell during exponential growth [ 14, 15]. The antigenically variable spirochete *Borrelia hermsii,* with approximately five genome copies per cell when grown in broth media to late log phase and 15 copies per cell when isolated from mice [ 16]. The aphid symbionts of *Buchnera* species have been reported to carry hundreds of genome copies [ 17, 18]. The extremely large bacteria of the *Epulopiscium* species contain large quantities of DNA at certain times in their growth cycle which may represent 1,000 copies of the genome per cell [ 19, 20], though the size of the genome and the number of genome copies per cell have not been determined. Finally, the large nitrogen-fixing bacterium *Azotobacter vinelandii* has been reported to have 40–80 genome copies per cell [ 21, 22]. Although the exact genome number per cell and distribution of genomic DNA into daughter cells remain to be determined for these phylogenetically distinct bacteria, it is likely that all or some of these species carry DNA representing more than one copy of their genome during growth. However, the question of genome copy number has not been explored in most bacterial species.

The rapid growth of bacterial cells depends on precise control of chromosomal replication and the faithful segregation of replicated chromosomes into daughter cells during cell division. All eubacteria examined thus far have a single origin of replication *(oriC)* per chromosome, from which initiation of DNA replication occurs and proceeds bi-directionally. Many bacterial origins which have been identified are located in the vicinity of the *dnaA* gene, whose product is required for DNA replication [ 3, 23, 24]. Even the *E. coli oriC* (K01789), which is linked to *gidA* (X01631), is believed to have had a translocation away from the ancestral position near *dnaA* (L10328)[ 25] **.** Alternatively, the *Coxiella burnetii oriC* has been mapped to the *gidA*/ *rpmH* region (U10529) [ 26], whereas Caulobacter crescentus and *Rickettsia prowazekii* origins are found in the *hemE*/RP001 region (U13664) of their chromosomes [ 27– 29]. Thus, the location of bacterial replication origins and genes associated with origins are not strictly conserved. Bacterial chromosomal origins are often characterized by binding sites for DnaA (AAC76725) and an AT rich region required for production of an open complex to which DNA polymerase can be recruited [ 3, 30, 31]. E. coli also has an over-representation of GATC methylation sites at *oriC* that are involved in regulating initiation of DNA replication [ 32– 34]. Across from *oriC* on a circular chromosome is the terminus which is characterized by several elements including a *dif* site (S62735), a DNA sequence recognized by the XerCD recombinase (POA8P6, POA8P8) which resolves chromosome dimers [ 35]. Replicated chromosomes must be correctly segregated into the daughter cells prior to cell division. Cytological experiments using fluorescent probes have revealed that newly replicated chromosomal DNA is rapidly segregated to opposite cell poles in several bacteria including *E. coli, B. subtilis,* and C. crescentus [ 36– 41].

Neisseria gonorrhoeae (the gonococcus, Gc) is the causative agent of the sexually transmitted disease gonorrhea, which was recorded to be a human disease as early as 5 B.C.[ 42]. This obligate human pathogen has evolved independently of most commonly studied Gram-negative organisms. Its survival in the human population is aided by the presence of multiple antigenic variation systems affecting surface-exposed structures, allowing it to evade the host immune response [ 43]. Compared to *E. coli,* little is known about Gc DNA replication, recombination and repair systems, and how they may be interconnected [ 43]. Gonococci have been shown to exist as a mixture of monococcal and diplococcal cells [ 44], which divide by partial constriction and septation at mid-cell, with subsequent division planes forming at right angles to each other [ 45, 46]. While the genome of the gonococcal strain FA1090 (AE004969) has been sequenced and is located on a single chromosome, the origin of replication has not been defined.

Gonococcal pilin antigenic variation is mediated by homologous recombination leading to a gene conversion event [ 47– 49]. Current models for pilin antigenic variation require two copies of the expressed pilin gene to be present in a cell, which would occur if there are multiple chromosomes per cell or transiently after DNA replication [ 43, 50]. Previous studies have shown that pilin antigenic variation is not linked to replication restart by PriA (YP_208491) [ 51]. While the replication-associated helicase, Rep (YP_207868), does play a role in pilin antigenic variation [ 52], gonococcal *rep* (AE004969) mutants do not show the replication delay phenotype of *E. coli rep* (M11055) mutants [ 53]. We therefore examined Gc DNA content to ask whether the high frequency gene conversion reactions underlying pilin antigenic variation could rely on multiple copies of the genome. Using flow cytometry and fluorescent microscopy, we show that gonococci contain a level of DNA content representing more than one genome copy per cell. Microarray and quantitative PCR analyses show that gonococci initiate bi-directional replication once per round of cell division, suggesting that the DNA content of the gonococcus reflects multiple completely replicated chromosomes and not a single chromosome with multiple replication forks. Quantitative PCR also demonstrated that the average genome copy number is three genomes per coccal unit. These results indicate that gonococci are polyploid, which may be necessary for efficient pilin antigenic variation and survival within the human host.

## Results

### DNA Content of Gonococci Measured by Flow Cytometry

**Figure 1:**
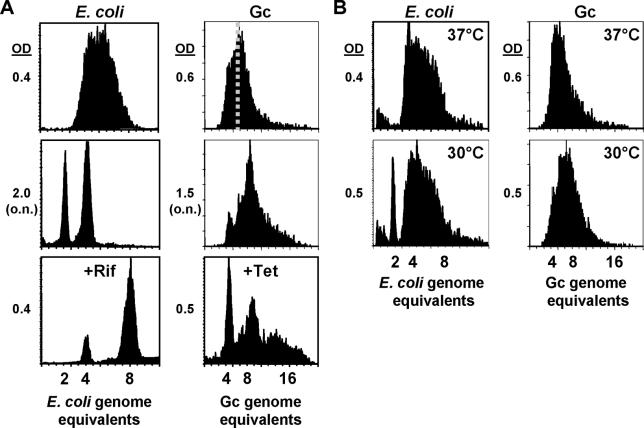
Flow Cytometry of Hoechst-Stained, Fixed Bacterial Cells For each histogram, the *x-*axis shows fluorescence levels, which indicate the amount of DNA content per particle counted. The *y-*axis shows counts, which indicate the number of fluorescing particles or cells. Culture optical densities (OD _600_) are listed to the left of each corresponding histogram. o.n., overnight culture. (A) E. coli (Column 1) and Gc (Column 2) growth curves under standard laboratory conditions. At mid-log phase, part of the culture was treated with rifampicin ( E. coli) or tetracycline (Gc). Genome equivalents were determined from the stationary phase and rif-treated E. coli and are shown on the *x-*axis. The dotted line represents the division line for sorting into higher and lower fluorescent populations. (B) E. coli and gonococcal cultures grown to mid-log phase at 37 °C or 30 °C. Genome equivalents were determined from stationary phase and rif-treated E. coli as in (A).

To begin to examine gonococcal DNA content, the well characterized properties of E. coli chromosomal DNA replication were used to create standards for flow cytometry ( Figure 1). Treatment of E. coli with certain antibiotics results in feedback inhibition of DNA replication initiation, leading to the completion of the current round of replication and resulting in fully replicated chromosomes [ 54]. In addition during stationary phase, cell division stops, initiation of DNA replication ceases, and active replication forks finish replication, resulting in integer numbers of fully replicated chromosomes per cell. E. coli that has been growing rapidly will have either two, four, or eight chromosomes in stationary phase, due to the presence of multiple pairs of replication forks at the time when cell division rates decline [ 4, 55, 56]. Stationary phase and rifampicin-treated E. coli cells were labeled with DNA-specific stains to produce cells carrying two, four, or eight genome equivalents per cell for flow cytometry ( Figure 1A). By comparison, untreated, exponentially growing E. coli cells showed a range of DNA content per cell that would be expected from cells with multiple replication forks that are at different stages in DNA replication and cell division ( Figure 1) [ 56]. Exponentially growing Gc cells also contained a broad range of DNA content as measured by flow cytometry ( Figure 1). The distribution of DNA content was similar in early, mid, and late log phase cultures ( Figure 1, and unpublished data). Since the Gc genome is 46.4% the size of the E. coli genome, we estimated that the majority of Gc cells in log phase contained two to six Gc genome equivalents of DNA ( Figure 1). There was a broad range of DNA content per cell observed for the two independent mid-log phase Gc cultures ( Figure 1 A and 1B) with the majority of cells carrying between four and six Gc genome equivalents. This level of DNA content per cell could reflect multiple chromosomes per cell, multiple pairs of replication forks on one or more chromosomes, amplification of a sub-chromosomal region, or the diplococcal form that is characteristic of Gc.

To begin to address whether Gc chromosomes can have multiple replication forks, cultures were enriched for fully replicated chromosomes. Growth of Gc into stationary phase resulted in autolysis as observed previously [ 57, 58], and the broad peak in the flow cytometry histogram for stationary phase cells indicates that replication only went to completion in a subset of Gc cells ( Figure 1A). However, the majority of cells in stationary phase contained about eight genome equivalents. Interpretation of the stationary phase results is problematic since only 10% of the cells remained viable, and this sub-population may not accurately represent the entire population. Treatment with either chloramphenicol (unpublished data) or tetracycline (Tet) produced intact non-growing Gc cells, which were enriched for completely replicated chromosomes ( Figure 1A). There were two major populations of cells in the Tet-treated cultures corresponding to four and eight Gc genome equivalents per cell ( Figure 1A). There was also a small population of cells showing fluorescence levels higher than eight, which either represents cell aggregates (see microscopic analyses below) or alternatively cells with higher number of replicated chromosomes. The antibiotic-treated and stationary phase gonococcal cultures revealed that exponentially growing Gc have two or four active pairs of replication forks per cell, but the number of chromosomes on which these forks exist could not be ascertained from these data.

In *E. coli,* a slower growth rate can be achieved by nutrient limitation in minimal media, and this slower growth results in less overlap of DNA replication cycles, fewer replication forks on the chromosome and an overall decrease in DNA content even at mid-log phase [ 56]. Gonococci are fastidious organisms which are unable to grow in minimal media, and the defined media used for gonococcal growth are quite complex and carry full nutrients [ 59, 60]. Therefore, to examine the effect of differing growth rate on Gc chromosomal content, lower temperatures were used to slow the growth rate. Gc cultures grown at 30 °C showed a doubling time of 90 min as opposed to 37 °C cultures which doubled in 60 min. There was no substantial change in the average DNA content per cell in the slower growing Gc cultures ( Figure 1B). As a control, E. coli cultures were grown at 37 °C, 30 °C, and 25 °C in rich media, resulting in 25-, 35-, and 50-min doubling times, respectively. The 25 °C and 30 °C cultures showed very similar patterns of DNA content ( Figure 1B and unpublished data). In these slower growing nutrient replete cultures (25 °C and 30 °C), a new population of E. coli cells with two genome equivalents of DNA was observed while the majority of cells had a similar range of DNA content as cells grown at 37 °C. It is possible that this new population of cells with two genome equivalents per cell represents cells between rounds of DNA replication. It is also possible that the reduced growth rate at lower growth temperatures results in slower replication fork progression, an alteration in the frequency of replication fork collapse, or an alteration in the efficiency of daughter chromosome resolution. Since the gonococcal DNA content profile is not substantially altered when growth is slowed and the doubling time increases by 1.5 fold, we propose that cell cycles do not overlap in gonococci.

### Microscopic Analysis of Gonococcal DNA Content

**Figure 2:**
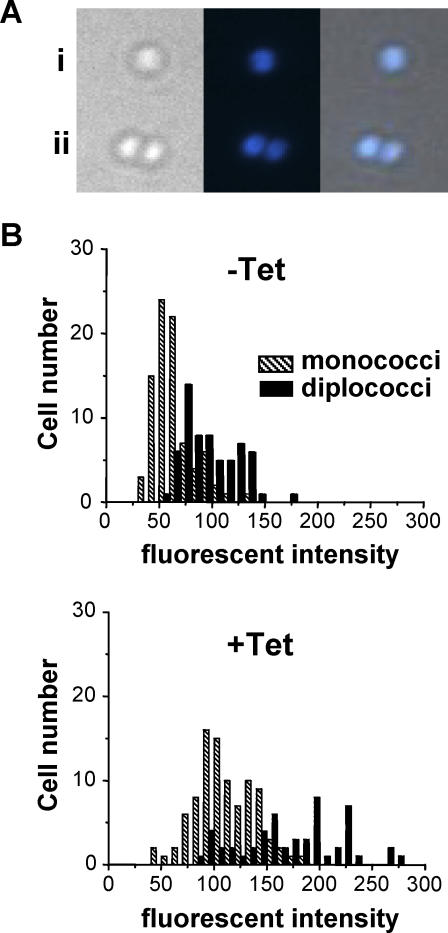
Microscopic Analysis of Gc DNA Content (A) Fluorescence microscopy of Hoechst-stained cells. Phase contrast, fluorescence, and merged images are shown for examples of each cell type observed. (i) monococci, (ii) diplococci. (B) Histogram of fluorescence intensities for exponentially growing and Tet-treated Hoechst-stained cells as determined from fluorescent micrographs.

Since Gc cultures contain a mixture of monococcal and diplococcal cell types, it was plausible that the populations of cells with different DNA content observed by flow analysis reflected the DNA content of monococcal and diplococcal forms. To explore the cellularity of Gc during growth, cells from cultures used for flow cytometry were examined by fluorescence microscopy ( Figure 2). About 90% of the particles from the exponentially grown and the Tet-treated Gc were either monococci or diplococci ( Figure 2A), while the remaining 10% contained greater than two coccal cell units. It was unclear whether these particles with more than two coccal units represent abnormal cell division products [ 61, 62], or are the result of cell aggregation. Therefore, particles containing more than two connected coccal units were excluded from further analyses. Tet treatment did not alter the ratio of monococci to diplococci ( Table 1).

**Table 1:**
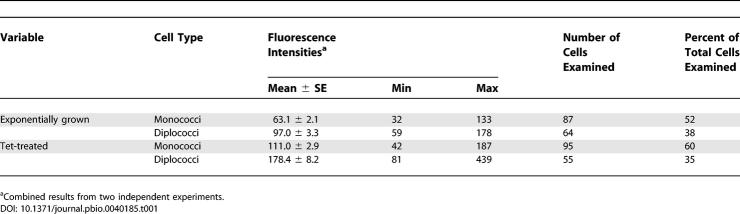
Analysis of DNA Content by Fluorescent Microscopy

The vast majority of gonococcal cells stained with DNA-specific dyes (both DAPI and Hoechst), showing that they contained chromosomal DNA, and essentially all diplococci were stained on both coccal units. This is the first indication that both halves of a diplococcus normally carry chromosomal DNA. Distinct nucleoids were not discernable using fluorescence microscopy due to the small size of the gonococcus (0.5 μm in diameter) and the fixation method used [ 63]. Slight condensation of nucleoids was observed with the Tet-treated gonococci, but again distinguishing distinct nucleoids was not possible at the level of resolution available.

The relative fluorescence intensity of both monococcal and diplococcal forms was measured and showed a range of DNA content ( Table 1). The mean relative fluorescence of monococci was less than that of diplococci, but was not half. We have no plausible explanation for this observation. The mean relative fluorescence of Tet-treated cells was 1.8-fold greater than that measured for exponentially growing cells. The difference in measured fluorescence intensity after Tet treatment most likely represents chromosomes caught at different stages of replication upon antibiotic treatment. If a chromosome had a single pair of active replication forks at the origin at the time of Tet treatment, then the DNA content would double. However, if a chromosome had multiple pairs of replication forks upon Tet treatment, the DNA content would more than double. Given that we observed an increase in DNA content after Tet treatment that was less than double suggests that the culture contained a mixture of cells between rounds of DNA replication and cells with a single pair of active replication forks per chromosome.

The DNA content distribution of cells examined by fluorescence microscopy ( Figure 2B) was comparable to the distribution observed in the flow cytometry analysis for the same culture ( Figure 1A), strongly suggesting that the presence of both monococci and diplococci contributes to the range of DNA content observed by flow cytometry. The majority of the exponentially grown and Tet-treated cells with lower DNA content were monococci, and the measured fluorescence intensities showed that monococci had a range of DNA content that was overlapping with, but on average less than, the DNA content of diplococci ( Figure 2B). To confirm that the microscopy and flow cytometry observations were consistent, exponentially grown Gc were sorted into lower and higher fluorescent populations as indicated in Figure 1A, and each sorted population was examined by fluorescence microscopy to determine the percentage of monococci and diplococci. In the population with lower DNA content, 73% of the cells were monococci, whereas the remainder were diplococci. The sorted population with the highest DNA content contained 37% monococci with the rest being diplococci or aggregates. These analyses confirm that one reason for the range of DNA content observed is a mixture of monococcal and diplococcal cells. By combining the microscopy, flow cytometry, and cell sorting data, we conclude that in growing cells, there are about three to six Gc genome equivalents per monococcus and four to ten Gc genome equivalents per diplococcus, split roughly into the two coccal halves.

### Gene Dosage Measurements Show Gonococci Are Polyploid

**Figure 3:**
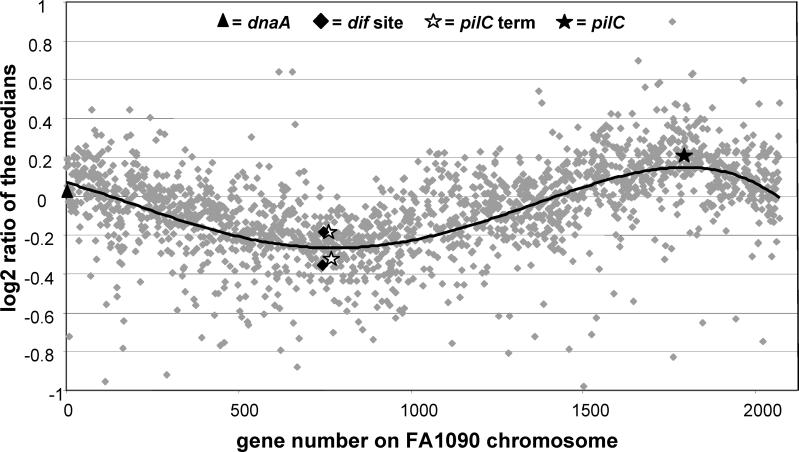
Marker Frequency Determined by Microarray Analysis Log2 ratio of the median differential hybridization signal of labeled DNA from untreated and Tet-treated exponentially growing cultures of N. gonorrhoeae. Gene number indicates the order of genes on the FA1090 genomic sequence starting with *dnaA*. The location of *dnaA*, *pilC1,* and the *pilC1* associated terminus are indicated by symbols along with the location of the genes surrounding the *dif* site. The trend line was produced using polynomial regression with an order of 4.

Bacterial replication origins have been identified in intergenic regions linked to *dnaA* [ 64– 66], *gidA, rpmH* [ 26], and *hemE/RP001* [ 27– 29]. In addition, the *oriC* for Neisseria meningitidis was predicted to be near *pilE* (X07731) by the *oriloc* computer program which predicts origins by analyzing GC di-nucleotide skew [ 67]. Using the *oriloc* program, the location of the gonococcal *oriC* was localized near the *pilC1* (Z50180) gene at position 1887 kilobases on the FA1090 genome sequence (G. Perriere, personal communication) in a region previously characterized in Gc [ 68]. To experimentally determine the location of the gonococcal *oriC* and measure marker frequency of origin and terminus sequences, microarray analysis was performed using genomic DNA obtained from both exponentially grown Gc and Tet-treated Gc (Tet-treated Gc are enriched for completely replicated chromosomes [ Figure 1A]). The genomic DNAs were fluorescently labeled and hybridized to a pan- *Neisseria* microarray, which contains PCR products representing every open reading frame (ORF) in the FA1090 genome (J. Davies et al. unpublished data). The results from triplicate hybridizations were averaged, and the relative intensity of the hybridization of DNA from exponentially grown Gc to DNA from Tet-treated Gc was plotted relative to the gene number on the genomic sequence ( Figure 3). Although there was substantial scatter to the data, a polynomial regression curve was fitted to the data to represent the average relative gene dosage around the chromosome. This analysis demonstrated that Gc has a single *oriC* represented by the single peak on the curve ( Figure 3). None of the origin-associated loci— *dnaA, parA/gidA,* or *hemE*—are located near the region of highest gene dosage. In contrast, the *dif* site is located at the region of the genome with the lowest gene dosage, confirming that this site is near the replication terminus. The *pilC1* gene is in the region with the greatest differential hybridization, and is also halfway across the circular chromosome from the *dif* site, suggesting that the origin region predicted by di-nucleotide skew is correct. Interestingly, none of the ORFs located near *pilC1* have been previously associated with bacterial replication origins ( Table S1). Moreover, the ratio of hybridization values of ORFs near the predicted origin versus those near the terminus was calculated to be 1.5:1.

**Figure 4:**
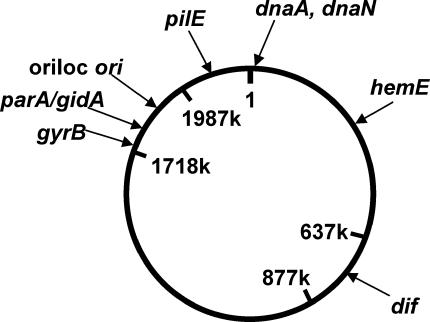
Diagram of the FA1090 Chromosome Quantitative PCR target sequences are shown, as well as putative origin sites, genes associated with bacterial origins, and the *dif* site which is linked to the terminus.

To confirm that the gene dosage between the origin and terminus was indeed less than two, quantitative PCR was used to measure the copy number of target sequences flanking the predicted origin and terminus ( Figure 4). To obtain total chromosomal DNA for PCR analysis, Gc were resuspended in water, boiled to release the DNA, and used directly in the PCR reaction [ 69]. For the exponentially growing cells, the number of coccal units contributing DNA to the PCR reactions was determined microscopically. As before, about 50% of the untreated Gc cells were monococci. Unfixed, Tet-treated Gc formed large aggregates making cell enumeration by light microscopy problematic. Therefore colony forming units (CFU) were used to normalize between the Tet-treated samples, which prevented a direct comparison between the growing and Tet-treated cells. Tet-treated Gc cells showed equivalent copies of each target sequence per CFU regardless of the location ( Table 2), which confirmed that Tet treatment enriched for completely replicated chromosomes. As expected, exponentially grown Gc had more copies of the origin-associated target sequences than the sequences near the terminus. The ratio of the copy number of origin-to-terminus-flanking sequences for growing Gc was measured to be 1.4:1 by quantitative PCR, which is consistent with the results obtained by microarray analysis.

**Table 2:**
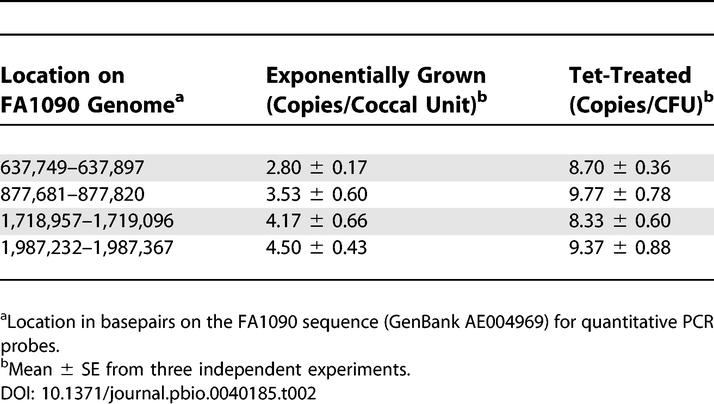
Gene Dosage Measured by Quantitative PCR

Quantitative PCR analysis also provided an independent measure of the DNA content of Gc cells. The copy number of sequences at the terminus is an indicator of the number of chromosomes independent of their replication status. The target sequences closest to the terminus were measured to have 2.8–3.5 copies per coccal unit in exponentially grown cells ( Table 2). Therefore, Gc have on average three chromosomes per monococcus and six chromosomes per diplococcus when growing in vitro. These quantitative PCR results support the flow cytometry and microscopy observations with exponentially grown cells, and taken together show that the gonococcus is polyploid during exponential growth.

## Discussion

Based upon this detailed analysis of the DNA content of *N. gonorrhoeae,* we conclude that gonococci have multiple complete chromosomes per coccal unit at all stages of growth. Flow cytometry, fluorescence microscopy and cell sorting results all indicate that monococci carry two to six Gc genome equivalents per cell and diplococci have four to ten Gc genome equivalents per cell. A slower growth rate did not substantially alter Gc DNA content while a new population of cells with DNA content equivalent to two fully replicated chromosomes was observed with E. coli ( Figure 1B). Gene dosage measurements by DNA microarrays and quantitative PCR revealed that only one initiation event occurs per chromosome per round of cell division, and there were no sub-genomic regions that were amplified relative to the rest of the genome. In addition, the copy number of terminus flanking sequences measured by quantitative PCR reveal that Gc have on average three genome copies per coccal unit. These findings are all consistent with the conclusion that gonococcal cells contain more than one genome copy in each coccal unit and are therefore polyploid.

We have determined that during exponential growth of Gc, a single pair of replication forks is present on each chromosome for each round of cell division by measuring the origin-to-terminus ratio via both microarray analysis and quantitative PCR. The ratio of origin-to-terminus sequences indicates the number of active replication forks present on a chromosome, and a single initiation event leads to a ratio of 2:1. A chromosome not undergoing DNA replication has a marker ratio of 1:1 as was measured for the Tet-treated Gc cultures ( Table 2). Assuming that replication of most origins in a cell are initiated simultaneously, as is the case with E. coli [ 1], multiple initiation events on a single chromosome would result in multiple pairs of replication forks and a ratio of 4:1 or 8:1 [ 70]. Asynchronous initiation of replication at the origins within a cell has been observed in certain E. coli mutants, which results in odd numbers of replication forks per chromosome with dispersed DNA content after antibiotic treatment [ 1]. Since Tet treatment of Gc produced a majority cells with four or eight genome equivalents ( Figure 1A), it appears that initiation usually occurs simultaneously at all origins within a cell. The measured origin-to-terminus ratio for exponentially growing Gc of 1.4 to 1.5:1 suggests that each population consists of a mixture of cells with fully replicated chromosomes and cells with a single pair of active replication forks on each chromosome. Therefore, the DNA content observed in Gc cells is not due to multiple initiation events on a single chromosome but reflects multiple fully replicated chromosomes.

From extensive studies of E. coli DNA replication, models and equations have been developed and tested in which the rate of replication, the time required to replicate the chromosome bi-directionally, and the time between completion of DNA replication and cell division have been calculated. Using the origin-to-terminus ratio and the doubling time of exponentially growing Gc, it is possible to calculate the time required to replicate the FA1090 chromosome (C period), the time between completion of replication and cell division (D period), and the rate of DNA replication. The measured ratio of origin-to-terminus copies (O/T) is 1.5, and the time between cell divisions (τ) is 60 min. The C period calculated from the equation O/T = 2 ^C/τ^ is 35 min [ 71]. The rate of chromosomal DNA replication was determined from the size of the chromosome and the calculated C period. Bearing in mind that replication is bi-directional, the rate of replication for Gc is calculated as being 513 base pairs per s. The D period can be calculated from the equation origins/cell = 2 ^(C+D)/τ^ [ 71]; however, we propose that there are a minimum of two chromosomes per coccal unit and have adjusted the equation accordingly, (origins/cell)/2 = 2 ^(C+D)/τ^. From quantitative PCR data, there are on average, four origins/cell during exponential growth of Gc ( Table 2), and the calculated D period is 25 min for each chromosome. While these estimates are informative for predicting cell cycle parameters, all of these calculated values need to be experimentally validated.

**Figure 5:**
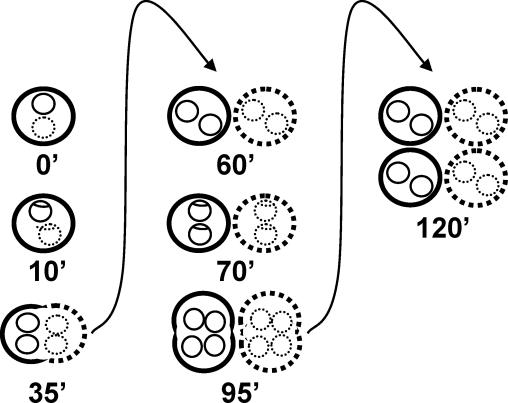
Model for Multiple Genomes per Monococcal Cell The proposed model has a monococcal cell undergoing DNA replication and cell division over time as indicated in min below each drawing. Segregation of chromosomes to promote homozygosity is demonstrated by the dotted chromosomal DNA.

Based upon these calculations, a model for Gc replication and cell division is proposed in which newly divided Gc carry two fully replicated chromosomes per monococcal cell, and a single round of replication occurs per chromosome per round of cell division. This replication produces monococcal cells carrying four chromosomes, which can be partitioned into the two daughter cells ( Figure 5). Since gonococci behave as haploid organisms, this suggests that cell division partitions identical copies of the genome into daughter cells. Further investigation of chromosome segregation dynamics will be required to directly test whether our model of two chromosomes replicating and segregating is correct, and whether gonococci are diploid homozygous or can also be heterozygous.

Most of the bacterial species which have been reported to be polyploid appear to have more than one copy of the genome per cell during exponential growth [ 15, 16, 18, 21, 22]. This is different from both E. coli and B. subtilis which appear to have a single copy of their genome per cell that replicates to form two complete copies when growth rates are slow [ 3, 5], and are diploid in the short window of time between the end of replication and cell division. In contrast, the gonococcus has more than one genome copy during all phases of growth. The data reported here indicates that Gc maintains at least two chromosome copies per coccal unit of a monococcal or diplococcal cell, which replicate in concert to four copies that are returned to two copies during cell division ( Figure 5). When the growth rate of E. coli and B. subtilis increases to outpace replication, these species carry multiple replication forks on the chromosome and therefore are partially polyploid for genes located near *oriC*. This partial polyploidy is different from gonococcal polyploidy, where there are a pair of replication forks on each chromosome but greater than two chromosomes per coccal unit. Bacteria that exhibit partial polyploidy due to fast growth do become polyploid when they enter stationary phase and stop growing. This conditional polyploidy is mechanistically distinct from the continual polyploidy of gonococci and other truly polyploid bacteria.

The bacterial species previously reported to carry multiple genome copies have not been examined to the same level of detail brought to these studies of N. gonorrhoeae. None of the previous studies examined replication fork dynamics nor did they account for localized DNA amplification. We presume that there are many other polyploid bacterial species, and it would be interesting to determine the genomic DNA content of other bacteria. Since only a subset of examined bacteria possess multiple genome copies at all stages of growth, it is possible that the evolution of polyploidy reflects a shared mechanistic basis. Based on comparisons between Gc and the other organisms previously reported to carry multiple genome copies, we suggest that one evolutionary force driving polyploidy could be DNA recombination. Polyploidy in D. radiodurans is suggested to aid in the DNA repair capabilities of this organism by promoting recombinational repair [ 72]. Gc lack a SOS response [ 73], and having multiple copies of the genome per cell may supplant the need for an inducible repair system. We have found that both the RecBCD and RecF-like homologous recombination pathways contribute to DNA repair in Gc [ 74]. It is interesting to note that both N. gonorrhoeae and D. radiodurans are coccal organisms, existing in diplococcal and tetracoccal forms, respectively. The similar polyploidy nature and cell morphology of these phylogenetically unrelated species suggests a possible linkage between these aspects of bacterial physiology, particularly since Gc has about two genomes within a coccal unit of a diplococcus, and *Deinococcus* has about four genomes within a coccal unit of a tetracoccus.

We initiated these studies to ask whether there might be more than one genome copy to allow for pilin antigenic variation. Gc possess one of the most potent diversity generation systems, driving pilin antigenic variation. Both B. hermsii, which is polyploid, and Borrelia burgdorferi express high frequency antigenic variation systems that appear mechanistically similar to the Gc pilin systems, with the major known difference being that the *Borrelia* carry the recombining genes on linear plasmids [ 75]. All of our current models for gonococcal pilin antigenic variation [ 43, 50] propose that two copies of the expressed pilin gene be present in a single cell. While two copies of the chromosome are transiently present immediately after DNA replication, the hybrid intermediate model also invokes loss of one chromosome during recombination [ 50]. If one chromosome is lost, only one intact chromosome would remain for chromosome segregation and cell division. While the hybrid intermediate model has experimental support [ 50], it is still unproven and the importance of multiple genome copies in antigenic variation remains untested. However, it is likely that polyploidy has a significant influence on gonococcal physiology, genetics, and pathogenesis.

## Materials and Methods

### Bacterial strains and growth conditions.

E. coli strain AB1157 was grown in Luria broth (LB) or on LB agar plates at 37 °C. N. gonorrhoeae strain FA1090Δ *pilE* was grown in GC liquid medium (GCL; 1.5% proteose peptone #3 [BD, Difco, Franklin Lakes, New Jersey, United States], 0.4% K _2_HPO _4_, 0.1% KH _2_PO _4_, 0.1% NaCl) with Kellogg supplements, [ 76] and 0.042% sodium bicarbonate at 37 °C with shaking, or on GC medium plates (GCB; BD, Difco) plus Kellogg supplements at 37 °C with 5% CO _2_.

### Growth curves.

For E. coli growth curves, overnight cultures of AB1157 were diluted 1:100,000 in LB plus 0.2% glucose in a baffle flask. Cultures were incubated at 25, 30, or 37 °C with shaking at 200 rpm. For rifampicin treatment, 5 ml of the 37 °C culture at OD _600_ = 0.5 was transferred to a 15-ml conical tube, 150 μg/ml rifampicin was added, and the treated culture was incubated in a rotor at 37 °C for 4 h. At each time point, (1) the OD _600_ was recorded; (2) 20 μl of culture was serially diluted in LB and plated for CFU per ml; (3) 10 μl of culture was examined using a hemacytometer to count the number of cells per ml; (4) 1 ml of culture was transferred to an Eppendorf tube, centrifuged at 10,000 rpm for 5 min, pelleted cells were washed with 500 μl of Tris-EDTA (TE), resuspended in 100 μl of ice-cold TE, and added to 900 μl of ice-cold 70 % EtOH to fix. Fixed cells were stored at 4 °C.

For gonococcal growth curves, 20- to 24-h-old colonies of FA1090Δ *pilE* on GCB plates were swabbed into GCL broth (OD _550_ = 0.05–0.15) and incubated for 16 h at 37 °C, rotating. The cultures were diluted 1:6 and incubated for 3 h at 37 °C or 6 h at 30 °C, rotating. The cultures were then diluted into 100 ml of GCL broth in a baffle flask to an OD _550_ = 0.05–0.1. The flask was incubated at 37 °C or 30 °C with shaking at 200 rpm. At OD _550_ = 0.4–0.5, 5 ml of culture was transferred to a 15-ml conical tube, Tet (2 μg/ml, Sigma, St. Louis, Missouri, United States) or chloramphenicol (5 ug/ml, Sigma) was added [ 77], and the culture was incubated for 90 min at 37 °C, rotating. At each time point, (1) the OD _550_ was recorded; (2) 20 μl of culture was serially diluted in GCL and plated for CFU per ml; (3) 10 μl of culture was examined using a hemacytometer to determine the number of coccal units per ml; (4) 1 ml of culture was transferred to an Eppendorf tube, centrifuged at 10,000 rpm for 5 min, pelleted cells were washed with 500 μl TE, resuspended in 100 μl ice-cold TE, and added to 900 μl ice-cold 70% EtOH to fix; (5) 1 ml of culture was pelleted as above, resuspended in sterile ddH _2_O, and placed in a boiling water bath for 15 min to release total DNA; (6) 1 ml of culture was pelleted as above and used to extract chromosomal DNA using the QIAamp DNA Mini kit (Qiagen, Valencia, California, United States). The yield of chromosomal DNA was lower from the Qiagen kit compared to the boiled samples as measured by quantitative PCR. Fixed cells were stored at 4 °C. Boiled samples and chromosomal DNA were stored at −20 °C.

### Flow cytometry.

Fixed bacterial cells were stained following the protocol by Bernander et al [ 78]. Essentially, fixed cells were diluted to 1 × 10 ^6^ cells/ml in Tris-MgCl buffer (10 mM Tris-Cl, [pH 7.4], 10 mM MgCl _2_) and incubated on ice for 10 min. An equal volume of 4′, 6-diamidino-2-phenylindole, dihydrochloride (DAPI; Molecular Probes, Eugene, Oregon, United States) or Hoechst No. 33258 (Sigma) in Tris-MgCl buffer was added to a final concentration of 5 μg/ml DAPI or 1 μg/ml Hoechst, and cells were incubated with stain for at least 15 min on ice before running through the flow cytometer. Stained cells were examined using a Beckman Coulter (Miami, Florida, United States) Epics Elite ESP flow cytometer with a water-cooled Innova Enterprise argon laser from Coherent (Santa Clara, California, United States) at 350 nm excitation or a LSRII cytometer (Becton Dickinson Biosciences, San Jose, California, United States) with a UV laser and a 440/40 bandpass filter. The cytometer was triggered on fluorescence such that only fluorescing particles were counted, and data was analyzed with FCS Express (De Novo Software, Ontario, Canada). For each sample, approximately 1 × 10 ^4^ particles were counted. The lowest flow rate possible was used, such that 20–40 particles per s were examined.

### Fluorescence microscopy.

Fixed bacterial cells were stained with Hoechst dye as described above. To obtain the majority of cells within the same focal plane, stained cells were pipetted onto a glass slide and allowed to dry at room temperature in the dark. The dried cells were covered with Fluoromount (Southern Biotechnology, Birmingham, Alabama, United States) containing 2.5 mg/ml propyl gallate (ICN Biomedicals, Inc., Costa Mesa, California, United States) to prevent photobleaching, and then a coverslip. Slides were examined with a Leica DMIRE2 microscope and analyzed using Openlab software (Agilent Technologies, Palo Alto, California, United States). Approximately 100 cells were examined for each sample. The percentage of monococci, diplococci, and multicocci was determined from phase contrast images. Fluorescence intensity for individual cells was determined using ImageQuant Version 5.0 software (Molecular Dynamics, Sunnyvale, California, United States).

### DNA microarray analysis.

One μg each of purified chromosomal DNA from the Qiagen kit obtained above for both untreated and Tet-treated Gc were suspended in a total volume of 38 μl of H _2_O, combined with 12 μg of random hexamers (Roche, Basel, Switzerland), and denatured 5 min, at 99 °C. Five μl of 10× Buffer 2 (New England Biolabs, Beverly, Massachusetts, United States)/5 μl of dNTP + aa-dUTP mix (0.5 mM dGTP, dATP, dCTP, 0.3 mM dTTP [Gibco, San Diego, California, United States], 0.2 mM aminoallyl-dUTP [Sigma])/4 μl of Klenow exo (New England Biolabs) was added and the reaction incubated 18 h at 37 °C. The reaction was stopped by adding 5 μl of 0.5 M EDTA. Free amines were removed using QIAquick PCR purification kit (Qiagen) following the manufacturer's instructions, except performing three wash steps. The samples were ethanol precipitated, resuspended in 5 μl of H _2_O, and 3 μl of 2.5% sodium bicarbonate (Sigma) was added to each. The resuspended samples were added to Alexa-Fluor 555 and 647 dyes (Molecular Probes) dissolved in 2 μl of high-quality DMSO (Sigma) and incubated in the dark at 25 °C for 1 h. The unincorporated dye was removed using QIAquick PCR purification kit again following the manufacturer's instructions, except performing three wash steps. The labeled samples were ethanol precipitated, resuspended in 4 μl of H _2_O, combined, added to 24 μl of hybridization solution (25% formamide, 5× saline sodium citrate [SSC], 0.1% SDS, 1 mg/ml salmon sperm DNA), and denatured at 95 °C for 5 min. The hybridization mixture was centrifuged (1 min at 14,000 × g) to collect condensation and applied to a microarray slide. Hybridization was performed in a humidified slide chamber at 42 °C for 16 h. The slide was washed as follows: in 2× SSC/0.1% SDS at 42 °C for 5 min, 0.1× SSC/0.1% SDS at room temperature for 10 min, 0.1× SSC at room temperature for 1 min, and 0.01× SSC for 10 s. The microarray was scanned using a ScanArray 4000XL confocal laser scanner and ScanArray Express software (Perkin Elmer, Wellesley, California, United States).

Data from two arrays yielding five to six readings per gene (each array contains three spots for each open reading frame) was used to compute the geometric means of the normalized fluorescent ratio. Spots were excluded due to low signal. The log2 ratio of the medians were averaged for each gene using Microsoft Excel software, and the averaged values were plotted against the gene number which corresponds to the order of genes in the sequenced FA1090 chromosome. Polynomial regressions of different orders were tested and the equation with the best empirical fit (order = 4) was used to create a trend line for the scatter plot.

### Quantitative PCR.

Primers and hybridization probes were from Roche Diagnostics. The sequences of primers and probes are listed in Table S2. PCR products specific for four locations around the gonococcal chromosome were generated by conventional PCR using the forward (FOR) and reverse (REV) primers for each location in a PTC-100 machine (MJ Research, Inc., Waltham, Massachusetts, United States) with purified gonococcal chromosomal DNA as a template. The reaction mixtures contained 25 μM each primer, 200 μM each deoxynucleotide (Gibco), 3 mM MgCl _2_, 1 U *Taq* DNA polymerase (Promega, Madison, Wisconsin, United States), and 1 ng of template DNA in 1× *Taq* reaction buffer (Promega).

PCR products were separated on 1% agarose (Gibco BRL)/ 1% NuSieve (FMC BioProducts, Rockland, Maryland, United States) gels and extracted from the gel using the QIAquick PCR Purification Kit (Qiagen). The elution was carried out with water. The molar concentrations of the PCR products that served as standard DNA in the LightCycler experiments were determined by gel analysis using Low Mass ladder (Gibco) and λ/BstEII (New England Biolabs) DNA markers and confirmed by UV spectroscopy.

Quantitative PCR experiments were performed in a LightCycler instrument from Roche Diagnostics [ 79]. Each 20 μl reaction contained 2 μl of standard or unknown DNA, 0.5 μM each FOR and REV primer, 0.2 μM each HYB1 and HYB2 probe, and MgCl _2_, plus reaction buffer containing polymerase provided with the LightCycler DNA Master Hybridization Probes Kit (Roche) and used following the manufacturers instructions. The target sequences at each chromosomal location listed in Table 2 were amplified with the FOR and REV primers. The accumulation of PCR product was monitored once per cycle during the amplification process, after the annealing phase, by measuring fluorescence signals emitted by the corresponding hybridization probes (HYB1 and HYB2). The ratio of the acceptor fluorophore (RED-640) to the donor fluorophore (FITC) fluorescence is proportional to the amount of PCR product. The LightCycler software version 3.5.3 was used to analyze quantitative PCR data (Roche). The fluorescence ratio versus cycle is plotted for the standard DNA and the unknowns measured in the same run. A threshold intersecting these curves in the exponential phase is applied, and the software automatically quantifies the number of copies of target sequence present based upon the standards.

## Supporting Information

Table S1 Genes Located in 39-Kilobase Region Containing the Predicted Replication Origin (50 KB DOC)

Table S2 Primers and Probes Used for Real-Time PCR Analysis (37 KB DOC)

### Accession Numbers

The National Center for Biotechnology (NCBI) ( http://www.ncbi.nlm.nih.gov) accession numbers for genes, specific DNA sequences, and proteins discussed in this paper are *E. coli oriC* (K01789), *E. coli gidA* (X01631), *E. coli dnaA* (L10328), *C. burnetii oriC* (U10529), *C. crescentus hemE* (U13664), E. coli DnaA (AAC76725), *dif* (S62735), XerCD recombinase (POA8P6, POA8P8), N. gonorrhoeae strain FA1090 (AE004969), N. gonorrhoeae PriA (YP_208491), N. gonorrhoeae Rep (YP_207868), *N. gonorrhoeae rep* (AE004969), *E. coli rep* (M11055), *N. meningitidis pilE* (X07731), and *N. gonorrhoeae pilC1* (Z50180).

## Abbreviations

CFU: colony forming unit
Gc: Neisseria gonorrhoeae
ORF: open reading frame
*oriC*: origin of replication
