# Supplementary material for: The Obligate Human Pathogen, Neisseria gonorrhoeae, Is Polyploid
Source: PLoS Biol. 2006 May 30;4(6):e185. doi: 10.1371/journal.pbio.0040185 (PMC1470461; doi:10.1371/journal.pbio.0040185)
Supplement: Supplementary file 1 [file pbio.0040185.st001.doc]

Table S1: Genes located in 39 kb region containing the predicted origin of replication.

| Gene# | Gene product |
| --- | --- |
| 1782 | DnaJ |
| 1784 | 23S rRNA |
|  | tRNA |
|  | tRNA |
| 1785 | 16S rRNA |
| 1789 | DnaK suppressor protein |
| 1790 | pyrroline-5-carboxylate reductase ( ProC ) |
| 1791 | probable lipoprotein |
| 1792 | conserved hypothetical protein |
| 1793 | PilT |
| 1794 | PilU |
| 1795 | conserved hypothetical protein |
| 1796 | hypothetical protein |
|  | (*oriloc* predicted origin) |
| 1797 | PilC1 |
| 1798 | 6-phosphogluconate dehydrogenase, decarboxylating |
| 1799 | 3-deoxy-D-manno-octulosonic-acid transferase ( KdtA ) |
| 1800 | conserved hypothetical protein |
| 1801 | probable transmembrane transport protein |
|  | tRNA |
| 1802 | UDP-N-acetylglucosamine 1-carboxyvinyltransferase (MurA) |
| 1803 | phosphoglycerate kinase |
| 1804 | BolA-YrbA family protein |
| 1805 | FtsX |
| 1806 | FtsE |
| 1807 | thioredoxin-related protein |
| 1808 | arsenate reductase ( ArsC ) |
| 1809 | EpiH/GdmH-related protein |
| 1810 | glutamyl-tRNA synthetase ( GltX ) |
| 1811 | hypothetical protein |
| 1812 | acetyltransferase, putative |
| 1813 | MutS |
| 1814 | glyceraldehyde 3-phosphate dehydrogenase |
