# Supplementary material for: The Obligate Human Pathogen, Neisseria gonorrhoeae, Is Polyploid
Source: PLoS Biol. 2006 May 30;4(6):e185. doi: 10.1371/journal.pbio.0040185 (PMC1470461; doi:10.1371/journal.pbio.0040185)
Supplement: Supplementary file 2 [file pbio.0040185.st002.doc]

Table S2: Primers and probes used for real-time PCR analysis

| Name | Position^a^ | Length (nt) | Sequence 5’→3’ |
| --- | --- | --- | --- |
|  |  |  |  |
| 637kFOR | 637749 | 17 | GAACGGCACGACTTCAG |
| 637kREV | 637897 | 16 | CAGGCGCGCTTGTTTG |
| 637kHYB1 | 637817 | 23 | GATTCTCGTATTGGGCGTGCGAC-**FITC** |
| 637kHYB2 | 637841 | 23 | **RED**-CTTCCATCTGGACGTATCGCTGC-**p** |
| 877kFOR | 877681 | 17 | GCAACGGATACGTCTTG |
| 877kREV | 877820 | 20 | GGGCAATACAGTTACTAAGG |
| 877kHYB1 | 877752 | 24 | CCGATAAACGAGCCGAAATCACTG-**FITC** |
| 877kHYB2 | 877777 | 23 | **RED**-CTTTCGTCCTGATGCGGCTTTTG-**p** |
| 1718kFOR | 1718957 | 16 | GGCGGATATCACATTG |
| 1718kREV | 1719096 | 16 | CCGATAGGGCGATATC |
| 1718kHYB1 | 1719045 | 22 | GTCAAGCTCGGAAAGCCCCTTC-**FITC** |
| 1718kHYB2 | 1719068 | 24 | **RED**-CCGTAAAGGCGGTATTCGAATAGG-**p** |
| 1987kFOR | 1987232 | 17 | CGCTGGGGAAGTATATG |
| 1987kREV | 1987367 | 17 | CAACTACGACTGCATTC |
| 1987kHYB1 | 1987308 | 27 | CACATCATAACCCAATAACGGAAAATC-**FITC** |
| 1987kHYB2 | 1987280 | 29 | **RED**-GCCAATTTTGCAAACAATTATTTCAAATG-**p** |

^a^ First nucleotide position of the oligonucleotide sequence in the FA1090 genome, GenBank entry (AE004969 )

^b^ FITC, fluorescein

^c^ RED, RED-640; p, 5’phosphate group
